# Alteration of Piezo1 signaling in type 2 diabetic mice: focus on endothelium and BK_Ca_ channel

**DOI:** 10.1007/s00424-024-02983-4

**Published:** 2024-07-02

**Authors:** Chae Eun Haam, Sooyeon Choi, Seonhee Byeon, Eun Yi Oh, Soo-Kyoung Choi, Young-Ho Lee

**Affiliations:** Department of Physiology, Yonsei University College of Medicine, 50 Yonseiro, Seodaemun-gu, Seoul, 03722 Korea

**Keywords:** Piezo1, Yoda1, Type 2 diabetes, BK_Ca_ channel, Vasorelaxation

## Abstract

Piezo1 mechanosensitive ion channel plays a important role in vascular physiology and disease. This study aimed to elucidate the altered signaling elicited by Piezo1 activation in the arteries of type 2 diabetes. Ten- to 12-week-old male C57BL/6 (control) and type 2 diabetic mice (db^−^/db^−^) were used. The second-order mesenteric arteries (~ 150 μm) were used for isometric tension experiments. Western blot analysis and immunofluorescence staining were performed to observe protein expression. Piezo1 was significantly decreased in mesenteric arteries of type 2 diabetic mice compared to control mice, as analyzed by western blot and immunofluorescence staining. Piezo1 agonist, Yoda1, concentration-dependently induced relaxation of mesenteric arteries in both groups. Interestingly, the relaxation response was significantly greater in control mice than in db^−^/db^−^ mice. The removal of endothelium reduced relaxation responses induced by Yoda1, which was greater in control mice than db^−^/db^−^ mice. Furthermore, the relaxation response was reduced by pre-treatment with various types of K^+^ channel blockers in endothelium-intact arteries in control mice. In endothelium-denuded arteries, pre-incubation with charybdotoxin, an Ca^2+^-activated K^+^ channel (BK_Ca_ channel) blocker, significantly attenuated Yoda1-induced relaxation in db^−^/db^−^ mice, while there was no effect in control mice. Co-immunofluorescence staining showed co-localization of Piezo1 and BK_Ca_ channel was more pronounced in db^−^/db^−^ mice than in control mice. These results indicate that the vascular responses induced by Piezo1 activation are different in the mesenteric resistance arteries in type 2 diabetic mice.

## Introduction

Diabetes mellitus (DM) is a chronic metabolic disease characterized by high blood sugar resulting from impaired insulin secretion, defective insulin action, or a combination of the two [14]. Vascular dysfunction, specifically the compromised endothelium-dependent relaxation, is linked to diabetes and is recognized as a crucial factor in the onset of vascular complications associated with diabetes [28]. Endothelial dysfunction is a central event in the pathogenesis of diabetes, and it greatly affects the development of future vascular complications [27]. However, the mechanisms underlying this damage remain incompletely understood. Therefore, it is crucial to comprehend the mechanisms responsible for endothelial dysfunction caused by diabetes mellitus and to identify treatments that can enhance or restore endothelial function to prevent diabetic vascular complications.

Mechanotransduction plays a pivotal role in vascular development, physiology, and disease [17]. Piezo1, a mechanically sensitive non-selective cation channel, was identified as an essential protein expressed in endothelial and vascular smooth muscle cells [6, 9, 16, 26]. The Piezo1 is considered a sensor for shear stress in vascular structures and is crucial for embryonic development [25]. Furthermore, Piezo1 plays important roles for vasculogenesis, valve morphogenesis, and the regulation of vascular tone [2, 24]. Interestingly, Piezo1 exerts atheroprotective effects by regulating nitric oxide (NO) release by the endothelium. Conversely, the activation of Piezo1 through high hydrostatic pressure not only disturbs the barrier function of lung endothelial cells but also leads to arterial remodeling under hypertensive conditions. Additionally, it induces a pro-atherogenic response when exposed to turbulent flow [8]. A previous study demonstrated that dysregulation of Piezo1 occurs in multiple blood lineages in patients with type 2 diabetes mellitus (T2DM). They also reported that elevated Piezo1 activity induces prothrombotic cellular responses in red blood cells, neutrophils, and platelets. Inhibition of Piezo1 protected against thrombosis in zebrafish genetic models and human blood samples, particularly in hyperglycemic conditions [36].

Although the significance of Piezo1 in vascular function has been studied, no reports have investigated the involvement of Piezo1 in diabetic vascular dysfunction. We hypothesized that activation of Piezo1 in mesenteric resistance arteries induces distinct vascular responses in control and diabetic mice, with differences in the underlying aspects and mechanisms governing Piezo1-induced responses between the two groups.

## Materials and methods

### Drugs

U46619 was obtained from Enzo Life Sciences (Farmingdale, NY, USA), and indomethacin was purchased from Calbiochem (Darmstadt, Germany). Dooku1 was acquired from Tocris Bioscience (Bristol, UK), and charybdotoxin (ChTX) was purchased from Alomone Labs (Jerusalem, Israel). All drugs and reagents, including Yoda1, were procured from Sigma-Aldrich (St. Louis, MO, USA).

### Experimental animals

Male C57BL/6 and db^−^/db^−^ (10 weeks) supplied by the Central Lab Animal Inc. (Seoul, Republic of Korea). The db^−^/db^−^ mice are characterized by a mutation in the leptin receptor gene, werve as a well-established animal model for type 2 diabetes. The mice were accommodated in a climate-controlled chamber with conditions set at a temperature of 22.0 ± 2°C, humidity maintained at 55 ± 5%, and a 12-h light/dark cycle, and had free access to food and tap water. All experiments were approved by the Animal Care and Use Committees at the Yonsei University College of Medicine (protocol number 2023-0016), and experimental procedures were performed according to the *Guide for the Care and Use of Laboratory Animals* published by the US National Institutes of Health (NIH publication no. 85-23, 2011).

### Tissue preparation

In all experiments, mice were euthanized using isoflurane inhalation. To confirm death, the mice were carefully checked for several signs, such as no response to toe pinch, no palpable heartbeat, and color change opacity in the eyes. We used mesenteric resistance arteries to investigate vascular function and to perform immunoreactive assays in the present study. The mesenteric resistance artery is one of the important proximal resistance vessels that contributes substantially to the peripheral resistance and widely used for studying vascular function. The small intestine, along with the attached vasculature, was swiftly excised and submerged in cold Krebs-Henseleit solution (KSH) (in mmol/L: NaCl 119, KCl 4.6, MgSO_4_ 1.2, KH_2_PO_4_ 1.2, CaCl_2_ 2.5, NaHCO_3_ 25, and glucose 11.1). The solution was continuously bubbled with a mixture of 95% O_2_ and 5% CO_2_. Second-order mesenteric arteries, approximately ~ 150 μm in diameter, were carefully isolated from surrounding fat and connective tissues. These vessels were then cut into 2- to 3-mm-long rings for use in isometric tension experiments. When necessary, endothelial denudation was achieved by perfusing the vessel segments with Triton X-100 (0.1%) for 10–15 s.

### Wire myography

Mesenteric artery segments were placed on a myograph chamber (620M, Danish Myotechnology, Aarhus, Denmark) using 25-μm wires and equilibrated for 20 min. The vessels stretched to a resting tension of ~ 2.5 mN. We used the normalization procedure to mimic physiological conditions and to stretch the mesenteric arterial segments to normalized internal circumference. To achieve optimal tension of mesenteric arteries, high K^+^ (70 mmol/L) solution-induced tension was measured in different tensions as previously reported [31, 35]. After 30 min of equilibration, the rings were stimulated twice with high K^+^ solution (in mmol/L: NaCl 53.6, KCl 70, MgSO_4_ 1.2, KH_2_PO_4_ 1.2, CaCl_2_ 2.5, NaHCO_3_ 25, and glucose 11.1) for 3−5 min at 10-min intervals prior to initiating the experiments.

### Experimental protocols

Upon inducing contraction with U46619 (10^−6^ M), concentration-dependent responses to Yoda1 (10^−7^–10^−5^ M, a selective Piezo1 activator) were recorded in mesenteric arteries of control and db^−^/db^−^ mice. To investigate whether the vascular endothelium plays a role in the vasodilatory mechanism of Yoda1, we measured the Yoda1 (10^−5^ M)-induced vasodilation in mesenteric artery rings pre-contracted with U46619 (10^−6^ M), both with or without vascular endothelium. When required, the endothelium was removed by perfusing with 0.1% Triton X-100 for 10–15 s. In control mice, the endothelium was considered denuded if acetylcholine (ACh, 10^−5^ M) induced less than 20% relaxation.

To assess whether Yoda1 (10^−5^ M)-induced vasodilation via the Piezo1, mesenteric arteries were pre-incubated with Dooku1 (5 × 10^−6^ M), a selective Yoda1 inhibitors, and GsMTx4 (2 × 10^−5^ M), a selective blocker of mechanosensitive ion channels.

To investigate the role of nitric oxide (NO), soluble guanylyl cyclase (sGC), and cyclooxygenase (COX) in Yoda1-induced vasodilation, arteries were pre-treated with the following specific inhibitors: N^ω^-Nitro-L-arginine (L-NNA, 5 × 10^−4^ M); nitric oxide synthase inhibitor, soluble guanylyl cyclase (sGC) inhibitor, 1H- [1,2,4]oxadiazolo [4,3,-a] quinoxalin-1-one (ODQ, 10^−5^ M); cyclooxygenase inhibitor, indomethacin (INDO, 10^−5^ M).

To characterize the K^+^ channels that mediate the relaxations to Yoda1, arteries were pretreated with the following agents: charybdotoxin (ChTX, 5 × 10^−8^ M), an BK_Ca_ channel blocker; triarylmethane-34 (TRAM-34, 10^−6^ M), an IK_Ca_ channel blocker; apamin (5 × 10^−7^ M), an SK_Ca_ channel blocker.

The equation used to calculate the degree of relaxation is as follows:

$$\textrm{Relaxation}\left(\%\right)=\left\{\left(\textrm{B}-\textrm{C}\right)/\textrm{B}-\textrm{A}\right\}\times 100$$

In the equation, *A* represents the resting tension of artery rings before pre-contraction with U46619 (10^−6^ M), *B* is the maximum contraction of artery rings after pre-contraction using U46619, and *C* is the contraction of the artery rings after the drug treatment.

### Western blotting

Mesenteric arteries were homogenized in ice-cold radioimmunoprecipitation assay (RIPA) (Thermo Fisher Scientific, Waltham, MA, USA, Cat# 89900) buffer containg protease and phosphatase inhibitor cocktail (Thermo Fisher Scientific, Cat# 78440). The protein concentrations in the tissue were determined using a bicinchoninic acid (BCA) protein assay (Thermo Fisher Scientific, Cat# 23227). Afterward, the loading samples were separated on 6–15% sodium dodecyl sulfate-polyacrylamide (SDS) gel and transferred to nitrocellulose membranes. Following a 1-h block with 5% (w/v) skim, the membranes were incubated overnight at 4 °C with primary antibodies against rabbit anti-Piezo1 (1:200; Alomone Labs, Cat# APC-087) and rabbit anti- KCNMA1 [K_Ca_ 1.1] (1:200; Alomone Labs, Cat# APC-151), followed by incubation with horseradish peroxidase-conjugated secondary antibody (1:5000; Santa Cruz Biotechnology, Cat# sc-2357). Protein bands were visualized using the SuperSignal West Pico plus chemiluminescent substrate (Thermo Fisher Scientific, Cat# 34557). In the case of phosphorylated proteins, membranes were stripped with Restore™ Western Blot Stripping Buffer (Thermo Fisher Scientific, Cat# 21059) and reprobed for total protein analysis. The density of β-actin bands from the same blots were used to normalize the target band for quantitative analysis.

### Immunofluorescence staining

Immunofluorescence staining was conducted to detect expression of Piezo1 and BK_Ca_ channels in the collected mesenteric arteries from both control and type 2 diabetic mice. The tissue was embedded in OCT (optimal cutting temperature) compound and snap frozen with liquid nitrogen (LN2) before being stored – 80 °C until further processing. Fresh-frozen section, 4 μm thick, were obtained using a cryostat. These sections were subsequently washed in Tris-buffered saline with tween20 (TBST) and then blocked in a bovine serum albumin (BSA) blocking buffer (5% (w/v) in TBST) for 1 h to minimize nonspecific binding. Following the blocking step, the sections were incubated overnight at 4 °C with a 1:100 dilution of the following primary antibodies: mouse-anti-CD31(Abcam, Cat# ab24590), rabbit anti-Piezo1 (Alomone Labs, Cat# APC-087), mouse anti-Piezo1 (Thermo Fisher Scientific, Cat# MA5-32876), and rabbit anti-KCNMA1 ([K_Ca_ 1.1] Alomone Labs, Cat# APC-151). Next, the sections were treated with a 1:100 dilution of secondary antibodies Alexa Fluor-488 conjugated donkey anti-mouse IgG (Therno Fisher Scientific, Cat# A-21202), Alexa Fluor-594 conjugated donkey anti-rabbit lgG (Therno Fisher Scientific, Cat# 21207), Alexa Fluor 594-conjugated donkey anti-mouse IgG (Thermo Fisher Scientific, Cat# A-32744), and Alexa Fluor 488-conjugated donkey anti-rabbit IgG (Thermo Fisher Scientific, Cat# A-32790) for 1 h at room temperature in the dark. The sections were mounted in VECTASHIELD Mounting Medium with DAPI (Vector laboratories, Peterborough, UK, Cat# H-120010). Fluorescence imaging of the stained sections was conducted using laser-scanning confocal microscopy (LSM710, Carl Zeiss, Germany).

### Quantification of colocalization

Pearson’s correlation coefficient (PCC) was employed for quantitative analysis of colocalization using the assistance of JACoP tool in Image J. The PCC is a statistical measure used to evaluate the overall association between two probes within an image, thus providing an indirect means to quantify their degree of colocalization. The sacle of the PCC ranges from − 1 to 1, where 1 represents strong colocalization, − 1 indicates negative colocalization, and 0 signifies no colocalization.

### Statistical analysis

The normal distribution of data was confirmed using Shapiro–Wilk’s test. Results are presented as mean ± SEM for the number of arteries derived from each distinct animal (*n*), except for pooled samples. Group comparisons were conducted using one-way or two-way ANOVA, followed by Bonferroni post-hoc tests for multiple comparisons. Statistical significance was defined as *P* values < 0.05. Statistical analysis was conducted using GraphPad Prism Version 10.0.0 (GraphPad Software Inc., San Diego, CA, USA).

## Results

### Expression of Piezo1 in mesenteric arteries

**Fig. 1:**
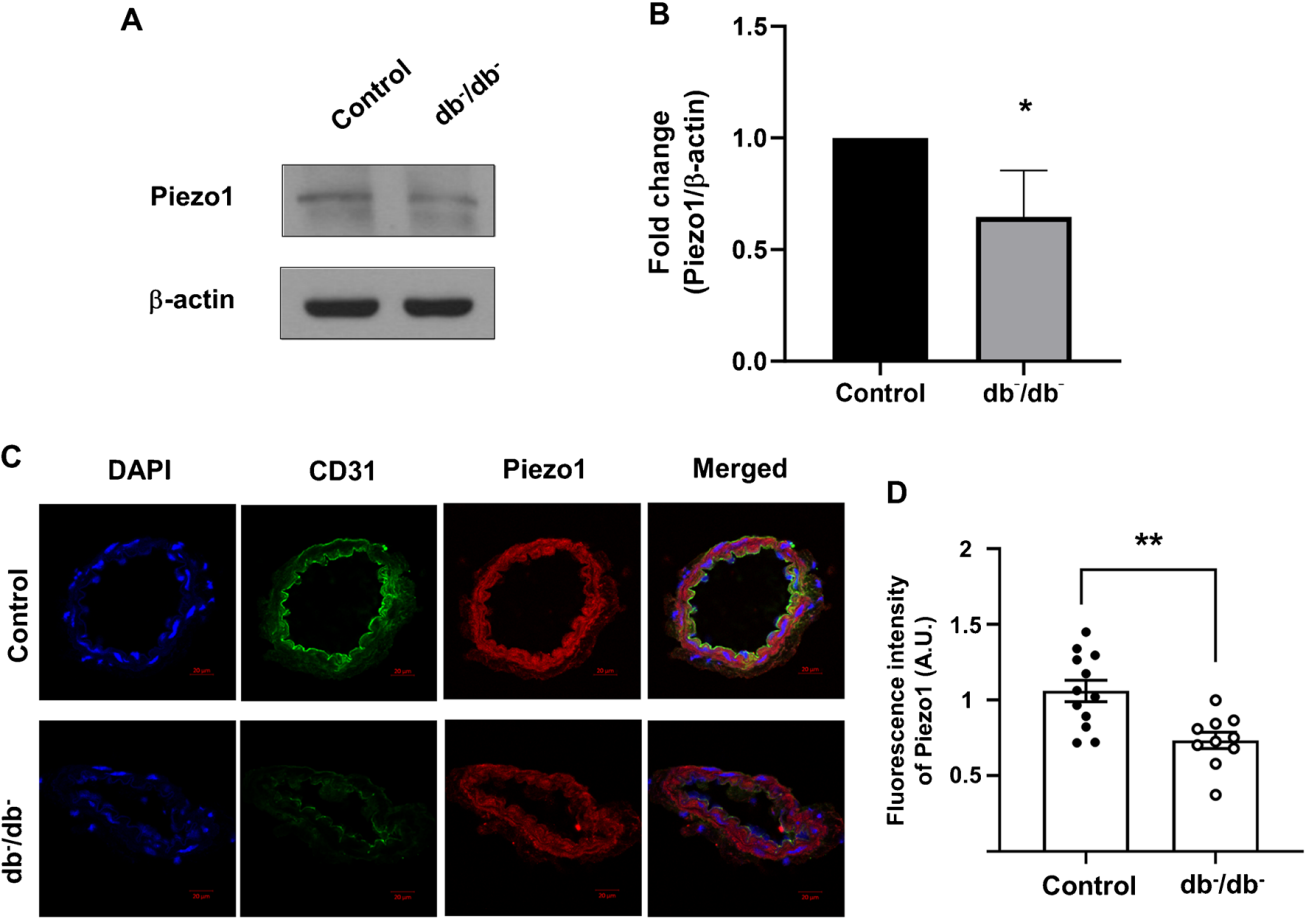
Piezo1 protein expression profiles in mesenteric arteries. Representative images (**A**) and summarized data (**B**) showing Piezo1 expression level and assessed by western blotting. β-actin was utilized as a loading control. Protein fold-change values are represented as the ratio between the Piezo1 protein band and the β-actin protein band. The data are presented as mean ± SEM (*n* = 5). ^*^p < 0.05, control vs. db^−^/db^−^. **C** Representative images of immunofluorescence staining of CD31 (an endothelial cell marker, green), Piezo1 (red) and DAPI (nuclei, blue). **E** Quantitative analysis of Piezo1 immunofluorescence. *n* = 12 for control, *n* = 10 for db^−^/db^−^

First, we examined whether Piezo1 is expressed in mesenteric arteries of both control and db^−^/db^−^ mice. Our western blot analysis showed a significantly lower expression level of Piezo1 in db^−^/db^−^ mice compared to control mice (Fig. 1A and B). To further visualize Piezo1 expression, we performed immunofluorescence staining with CD31 (green) and Piezo1 (red). The fluorescence signal of Piezo1 (red) was weaker in db^−^/db^−^ mice compared to control mice. Additionally, the expression of CD31 (green), serving as an endothelial cell marker, was also reduced in the mesenteric arteries of db^−^/db^−^ mice (Fig. 1C).

### Yoda1-induced vasodilation and involvement of endothelium

**Fig. 2:**
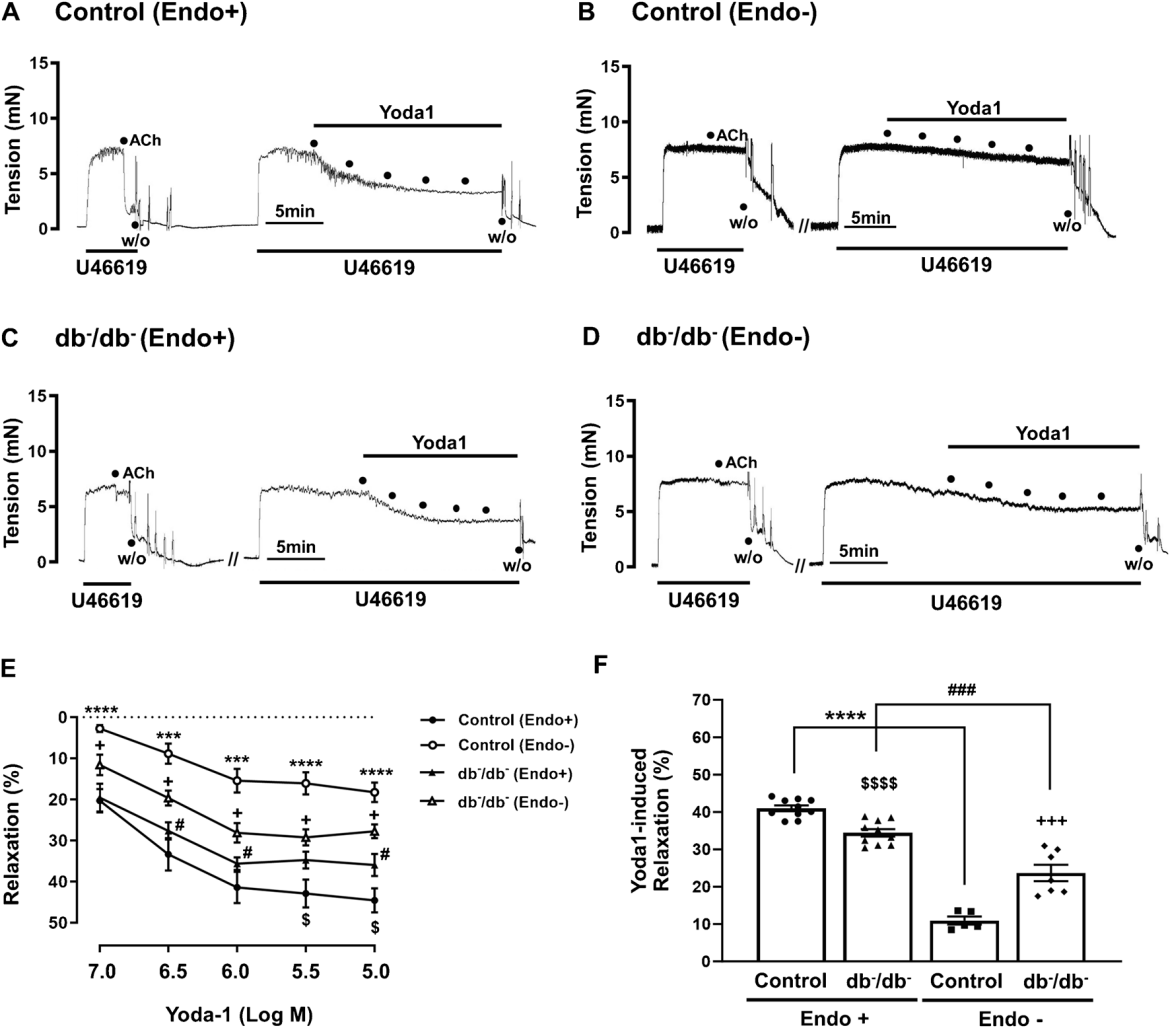
Effect of Yoda1 on vascular contractility in mesenteric arteries. Representative recording traces showing the effects of Yoda1 (10^−7^–10^−5^ M) in endothelium-intact (Endo+) and endothelium-denuded (Endo−) mesenteric arteries of control (**A**, **B**) and db^−^/db^−^ mice (**C**, **D**). Summarized graph (**E**) showing the relaxation responses caused by Yoda1 in mesenteric arterial rings. Summarized data (**F**) of Yoda1(10^−5^ M)-induced relaxation with endothelium (Endo+), and without endothelium (Endo−). **A**–**E** Relaxation (%) indicates the percentage of U46619 (10^−6^ M)-induced contraction. Results are presented as means ± SEM; *n* = 7 for control (Endo+), *n* = 7 for control (Endo−), *n* = 8 for db^−^/db^−^ (Endo+), *n* = 7 for db^−^/db^−^ (Endo−). ^***^p < 0.001, ^****^p < 0.0001, control (Endo+) vs. control (Endo−), ^#^*p* < 0.05, db^−^/db^−^ (Endo+) vs. db^−^/db^−^ (Endo−), ^$^*p* < 0.05, control (Endo+) vs. db^−^/db^−^ (Endo+), ^+^*p* < 0.05, control (Endo−) vs. db^−^/db^−^ (Endo−). **F** *n* = 10 for control (Endo+), *n* = 10 for db^−^/db^−^ (Endo+), *n* = 5 for control (Endo−), *n* = 7 for db^−^/db^−^ (Endo−). ^****^p < 0.0001, control (Endo+) vs. control (Endo−), ^###^*p* < 0.001, db^−^/db^−^ (Endo+) vs. db^−^/db^−^ (Endo−), ^$$$$^*p* < 0.0001, control (Endo+) vs. db^−/^db^−^ (Endo+), ^+++^*p* < 0.001, control (Endo−) vs. db^−^/db^−^ (Endo−). ACh, acetylcholine; W/O, wash out

To explore the impact of Piezo1 activation, we assessed the effect of Yoda1, a selective Piezo1 activator, on vascular contractility in isolated mesenteric artery segments from both control and db^−^/db^−^ mice. Yoda1 was administered at concentrations ranging from 10^−7^ to 10^−5^ M. Activation of the Piezo1 by Yoda1 (10^−7^–10^−5^ M) induced a concentration-dependent relaxation response in both control (Fig. 2A) and db^−^/db^−^ mice (Fig. 2C). Notably, this relaxation response was attenuated in db^−^/db^−^ mice compared to control mice (Fig. 2A and C). To assess the contribution of endothelium to Yoda1-induced relaxation, we performed experiments using endothelium-intact and endothelium-denuded artery rings. Yoda1-induced relaxation was significantly decreased in endothelium-denuded arteries in control (Fig. 2A and B) and db^−^/db^−^ mice (especially at concentrations of 10^−6.5^ M, 10^−6^ M, and 10^−5^ M, Fig. 2C and D). Interestingly, the removal of the endothelium decreased relaxation responses induced by Yoda1, and this effect was more pronounced in control mice than in db^−^/db^−^ mice (Fig. 2B and D). Endothelium-intact or endothelium-denuded arteries were pre-incubated with U46619 (10^−6^ M) and subsequently treated with Yoda1 at a concentration of 10^−5^ M, which elicited the maximum vasodilation effect. Yoda1 (10^−5^ M) induced relaxation responses in endothelium-intact arteries in both groups. Consistent with the concentration-response results, the relaxation activity caused by Yoda1 was greater in control mice than in db^−^/db^−^ mice (Fig. 2E). Endothelium-denuded arteries significantly reduced Yoda1-induced relaxation responses in both groups. Interestingly, endothelial removal had a greater effect in control mice (Endo+: 40.9 ± 0.8% vs. Endo−: 10.9 ± 1.1%, approximately 30% of reduction) than in db^−^/db^−^ mice (Endo+: 34.4 ± 1.0% vs. Endo−: 23.7 ± 2.2%, approximately 10% of reduction). Treating the vessels with Yoda1 (10^−7^–10^−5^ M), either with or without endothelium, had no effect on basal tension (data not shown). The vehicle, dimethyl sulfoxide (DMSO, 0.015%–0.150%), had no effect on U46619-induced precontracted rings when compared to treatment with Yoda1 (data not shown).

### Effects of Dooku1 and GsMTx4 on Yoda1-caused relaxation

**Fig. 3:**
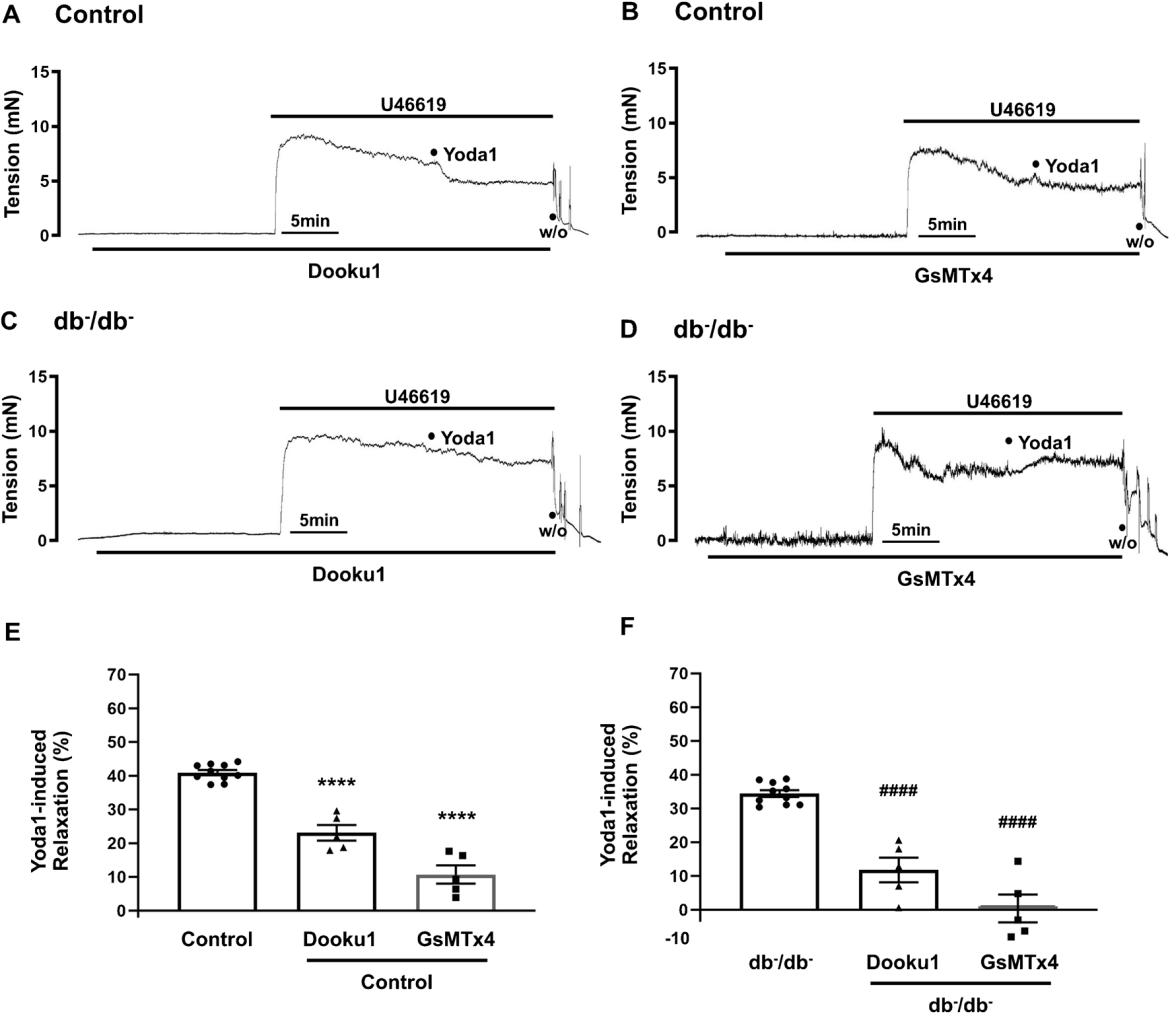
Effects of Dooku1 and GsMTx4 on Yoda1-induced relaxation. Representative traces showing the effects of Yoda1 (10^−5^ M) in the pretreatment of Dooku1 (5 × 10^−6^ M) or GsMTx4 (2 × 10^−5^ M) in mesenteric arteries of control (**A**, **B**) and db^−^/db^−^ mice (**C**, **D**). Summarized data (**E**, **F**) of Yoda1-induced relaxation (%) with or without Dooku1 and GsMTx4. Relaxation (%) represents the percentage of U46619 (10^−6^ M)-induced constriction. Results are presented as means ± SEM; *n* = 5 for control (Dooku1), *n* = 5 for control (GsMTx4), *n* = 5 for db^−^/db^−^(Dooku1), *n* = 7 for db^−^/db^−^ (GsMTx4). ^****^p < 0.0001 vs. control, ^####^ *p* < 0.0001 vs. db^−^/db^−^. W/O, wash out

As illustrated in Fig. 3E and F, Yoda1-induced vasodilation was measured at 40.9 ± 0.8% in control mice and 34.4 ± 1.0% in db^−^/db^−^ mice. Pre-treatment with Dooku1 (a selective inhibitor of Yoda1) and GsMTx4 (mechanosensitive channel-selective inhibitor) significantly reduced Yoda1-induced relaxation in both control (23.1 ± 2.3% and 10.7 ± 2.7%, respectively, Fig. 3A and B) and db^−^/db^−^ mice (11.8 ± 3.6% and 0.4 ± 4.1%, respectively, Fig. 3C and D). These findings demonstrate that Yoda1 exhibits specificity for the Piezo1 channel (Fig. 3). The vehicle, DMSO (0.1%), had no impact on U46619-induced contracted rings when compared to treatment with Yoda1 (data not shown).

### Effects of L-NNA, ODQ, and INDO on Yoda1-caused relaxation

**Fig. 4:**
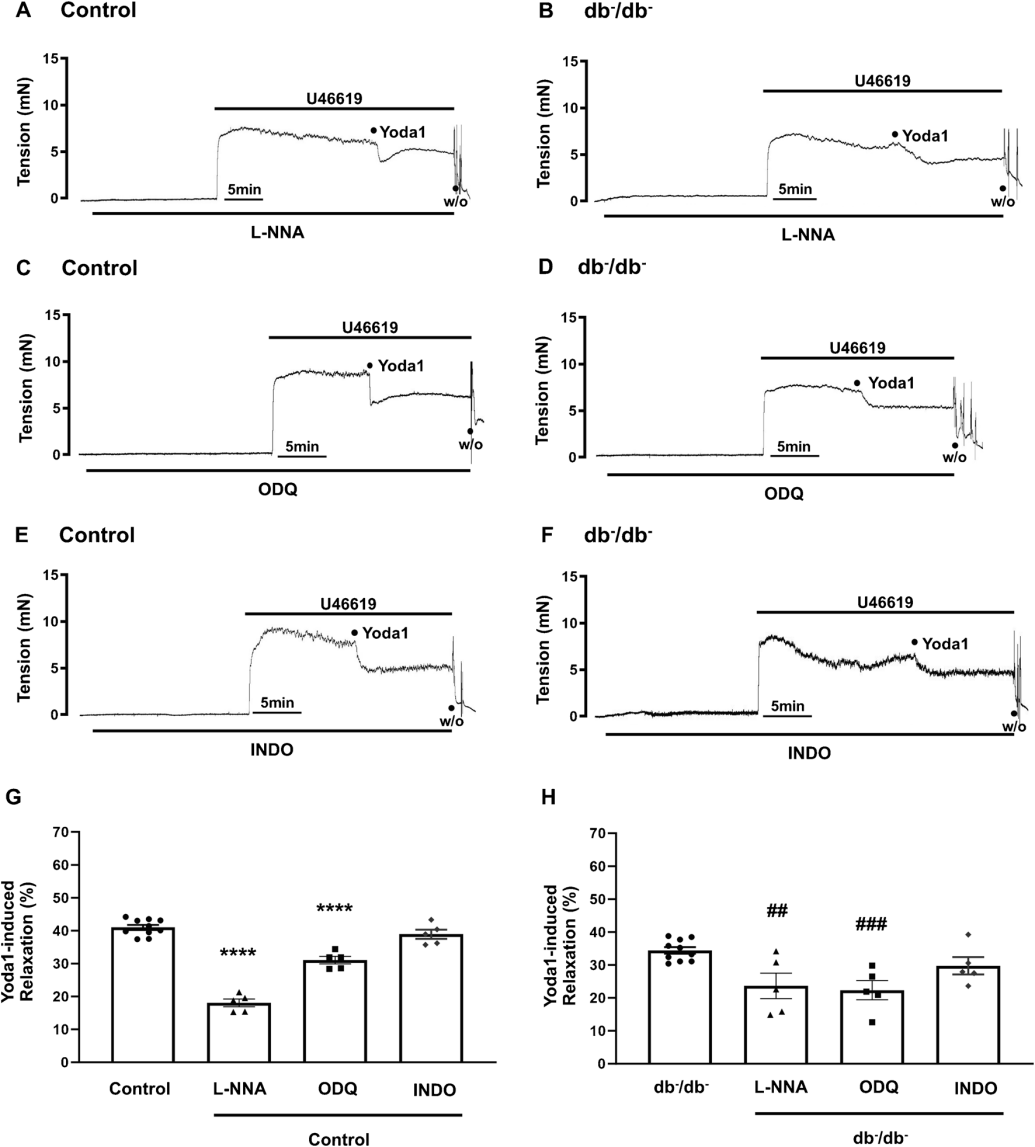
Effects of L-NNA, ODQ, and INDO on Yoda1-caused relaxation. Representative original traces of Yoda1-induced relaxation with L-NNA (**A**, **B**), ODQ (**C**, **D**), or INDO (**E**, **F**) in control and db^−^/db^−^ mice. The data in **G** and **H** represent mean ± SEM values of Yoda1-induced relaxation observed in the control (**G**) and db^−^/db^−^ mice (**H**). *n* = 5 for control (L-NNA), *n* = 5 for db^−^/db^−^ (L-NNA), *n* = 5 for control (ODQ), *n* = 5 for db^−^/db^−^ (ODQ), *n* = 5 for control (INDO), *n* = 5 for db^−^/db^−^ (INDO). ^****^p < 0.0001 vs. control, ^##^*p* < 0.01, ^###^*p* < 0.001 vs. db^−^/db^−^. L-NNA, N^ω^-Nitro-L-arginine; ODQ, 1H-[1,2,4]oxadiazolo [4,3,-a] quinoxalin-1-one; INDO, indomethacin; W/O, wash out

In both groups, pre-treatment with N^ω^-Nitro-L-arginine (L-NNA), a nitric oxide synthase inhibitor, significantly decreased Yoda1-induced relaxation compared to the untreated group (Fig. 4A and B). In control group, the relaxation response decreased from 40.9 ± 0.8 to 18.1 ± 1.2% after pre-treatment with L-NNA. In db^−^/db^−^ group, the relaxation response decreased from 34.4 ± 1.0 to 23.6 ± 3.9% following pre-incubation with L-NNA. Similarly, pre-incubation with ODQ, a soluble guanylate cyclase (sGC) inhibitor, also resulted in a marked reduction in Yoda1-caused relaxation in both groups (Fig. 4C and D). In control group, the Yoda1-induced relaxation response decreased from 40.9 ± 0.8 to 31.0 ± 1.1% following pre-treatment with ODQ. In db^−^/db^−^ group, the Yoda1-induced relaxation response decreased from 34.4 ± 1.0 to 29.7 ± 2.6% after pre-treatment with ODQ. However, pre-treatment with indomethacin (INDO), a cyclooxygenase (COX) inhibitor, showed no statistically significant difference in Yoda1-caused relaxation in both groups (Fig. 4E and F).

### Effects of IK_Ca_ and SK_Ca_ channel blockers on Yoda1-caused relaxation

**Fig. 5:**
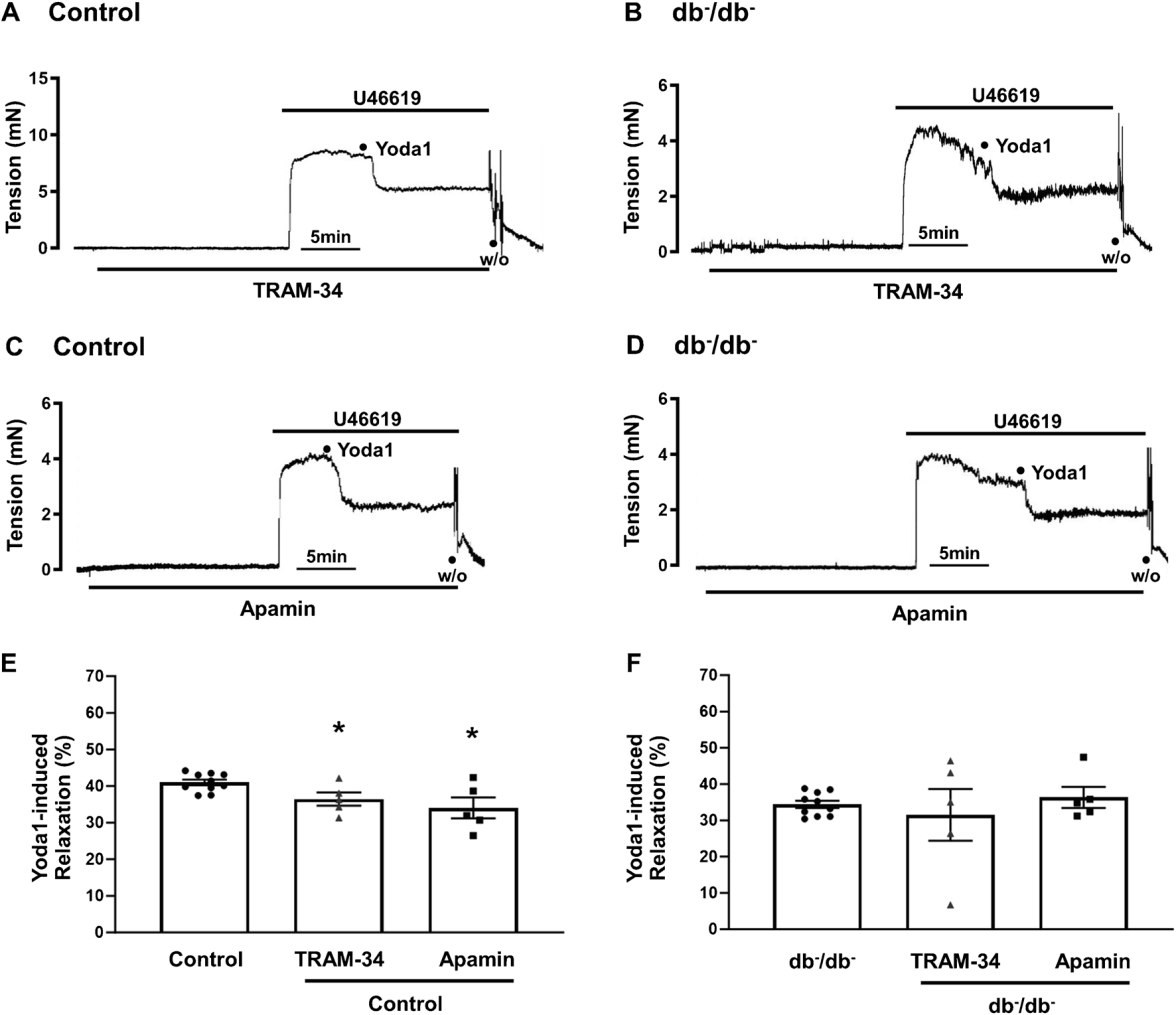
Effects of IK_Ca_ and SK_Ca_ channel blockers on Yoda1-induced relaxation. Representative traces showing the relaxation induced by Yoda1 (10^−5^ M) after pre-treatment with TRAM-34 (**A**, **B**) and apamin (**C**, **D**) in control and db^−^/db^−^ mice. The data in **E** and **F** represent the mean ± SEM values of Yoda1-induced relaxation observed in the experiments. *n* = 5 for control (TRAM-34), *n* = 5 for db^−^/db^−^ (TRAM-34), *n* = 5 for control (apamin), *n* = 5 for db^−^/db^−^ (apamin). ^*^p < 0.05 vs. control, ^#^*p* < 0.05 vs. db^−^/db^−^. TRAM-34, triarylmethane-34; IK_Ca_, intermediate-conductance Ca^2+^-activated K^+^ channel; SK_Ca_, small-conductance Ca^2+^-activated K^+^ channel; W/O, wash out

Our study explored whether IK_Ca_ and SK_Ca_ channels are involved in Yoda1-caused relaxation. In control mice, the relaxation activity to Yoda1 was modestly inhibited in the presence of TRAM-34 (10^−6^ M), an IK_Ca_ channel blocker, and apamin (5 × 10^−7^ M), an SK_Ca_ channel blocker. Yoda1-induced relaxation decreased from 40.9 ± 0.8 to 36.4 ± 1.8% after pre-treatment with TRAM-34 (Fig. 5A). The relaxation (%) to Yoda1 before and after apamin treatment was determined to be 40.9 ± 0.8% and 34.0 ± 2.8%, respectively (Fig. 5C). However, in db^−^/db^−^ mice, TRAM-34 and apamin did not affect Yoda1-caused relaxation (Fig. 5B and D).

### Effect of blocking BK_Ca_ channel on Yoda1-caused relaxation

**Fig. 6:**
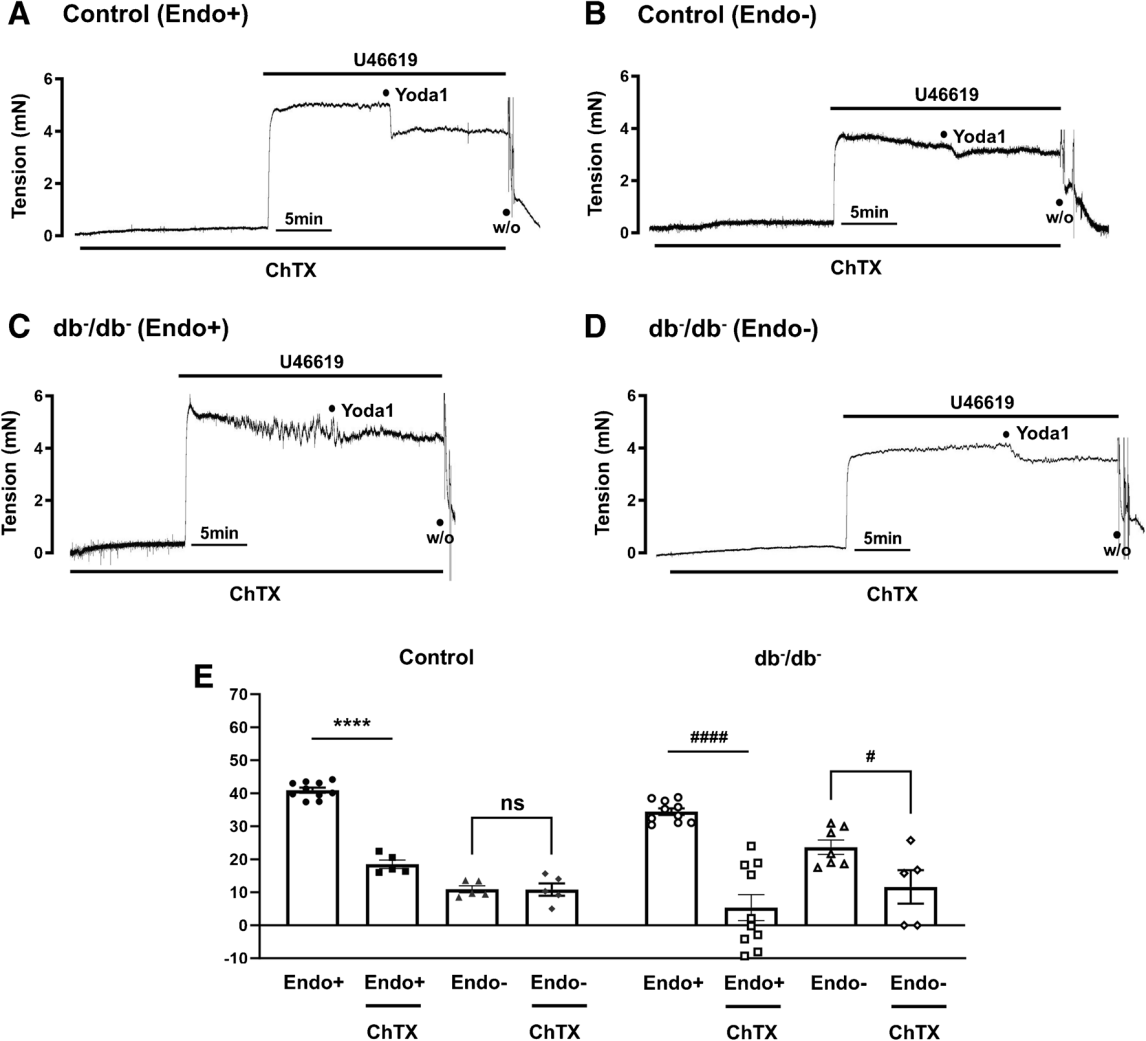
Effect of BK_Ca_ channel blocker on Yoda1-induced relaxation. Representative traces showing the relaxation induced by Yoda1 (10^−5^ M) after pre-treatment with ChTX (5 × 10^−8^ M) in endothelium-intact and endothelium-denuded mesenteric arteries of control (**A**, **B**) and db^-^/db^-^ mice (**C**, **D**). The data in **E** are presented as mean ± SEM values of Yoda1-induced relaxation observed in experiments. *n* = 10 for control (Endo+), *n* = 5 for control (Endo+) with ChTX, *n* = 5 for control (Endo−), *n* = 5 for control (Endo−) with ChTX, *n* = 10 for db^−^/db^−^ (Endo+), *n* = 10 for db^−^/db^−^ (Endo+) with ChTX, *n* = 7 for db^−^/db^−^ (Endo-), *n* = 5 for db^−^/db^−^ (Endo−) with ChTX. ns, no significant difference. ^****^p < 0.0001, control (Endo+ and ChTX−) vs. control (Endo+ and ChTX+), ^#^*p* < 0.05, db^−^/db^−^ (Endo− and ChTX−) vs. db^−^/db^−^ (Endo− and ChTX+), ^####^*p* < 0.0001, db^−^/db^−^ (Endo+ and ChTX−) vs. db^−^/db^−^ (Endo+ and ChTX+). ChTX, charybdotoxin; BK_Ca_, large-conductance Ca^2+^-activated K^+^ channel; W/O, wash out

To confirm the involvement of the BK_Ca_ channel in Yoda1-caused relaxation, we conducted studies using both endothelium-intact and endothelium-denuded mesenteric arteries. In control mice, pre-treatment with charybdotoxin (ChTX, 5 × 10^−8^ M), a BK_Ca_ channel blocker, significantly reduced Yoda1-induced relaxation in endothelium-intact arteries (non-treated: 40.9 ± 0.8%, ChTX-treated: 18.5 ± 1.3%; Fig. 6A). However, pre-treatment with ChTX did not affect Yoda1-induced relaxation in endothelium-denuded arteries in control mice (Fig. 6B). In contrast, Yoda1-induced relaxation was significantly inhibited by ChTX, not only in endothelium-intact arteries but also in endothelium-denuded arteries in db^−^/db^−^ mice. In endothelium-intact arteries in db^−^/db^−^ mice, pre-treatment with ChTX reduced Yoda1-induced relaxation from 34.4 ± 1.0 to 12.4 ± 3.9% (approximately 22% reduction, Fig. 6C). In endothelium-denuded arteries in db^−^/db^−^ mice, pre-treatment with ChTX reduced Yoda1-induced relaxation from 23.7 ± 2.2 to 11.3 ± 5.1% (approximately 12% reduction, Fig. 6D).

### Co-localization of Piezo1 and BK_Ca_ channel in mesenteric arteries

**Fig. 7:**
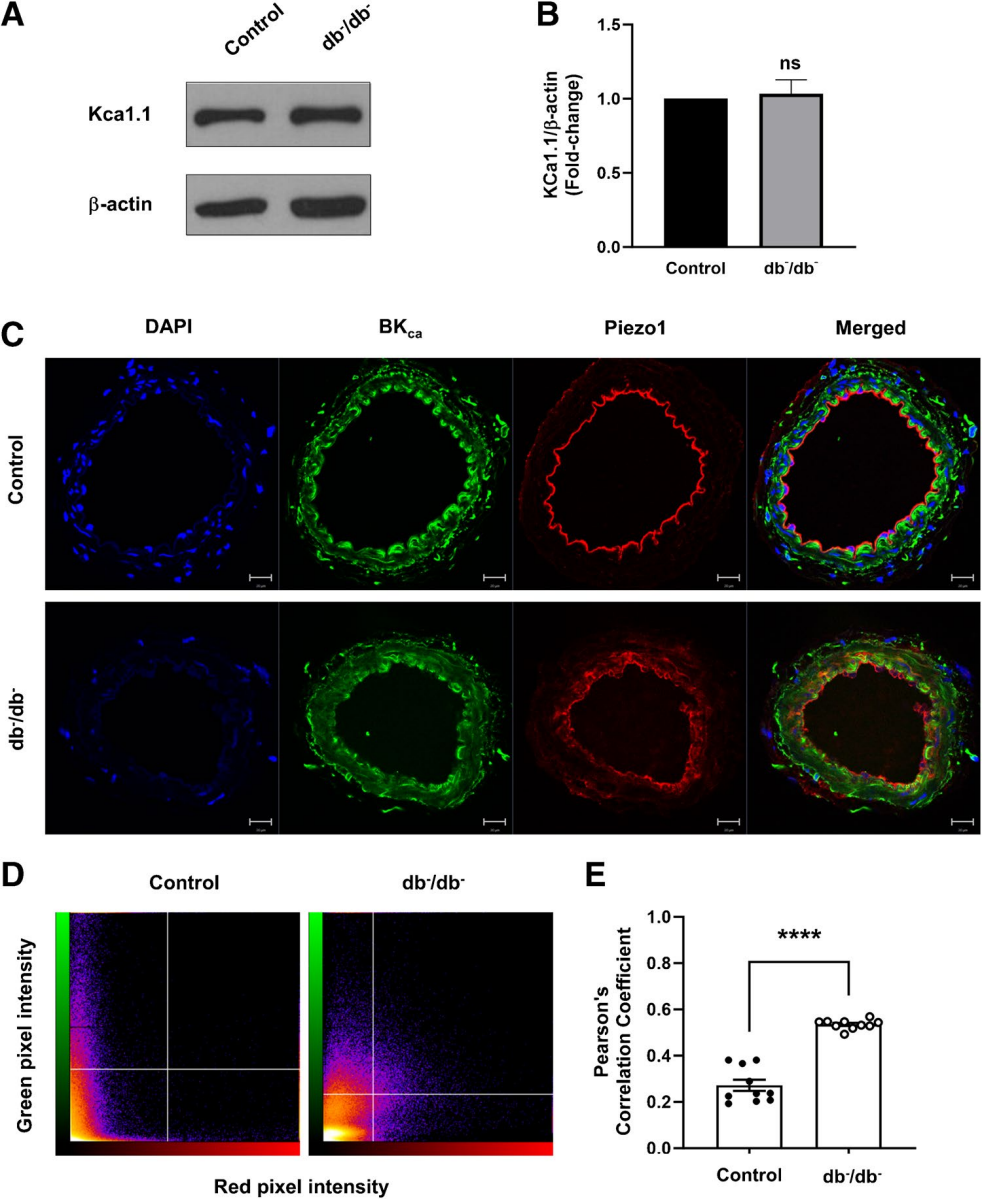
Co-localization of Piezo1 channels and BK channels in mesenteric arteries. Representative western blot (**A**) and semi-quantitative analysis (**B**) of protein level of KCa1.1 (also called BK_Ca_) in mesenteric arteries of control and db^−^/db^−^ mice. Protein expression was normalized to β-actin and expressed as fold change relative to control. Results are presented as mean ± SEM (*n* = 6). Representative images (C) of co-immunofluorescence staining of BK_Ca_ (green), Piezo1 (red), and DAPI (nuclei, blue). Scatter plot (**D**) of intensity level in each channel; green is shown on the *x*-axis and red is shown on the *y*-axis. The degree of co-localization was quantified and compared using Pearson’s correlation coefficient (**E**). Results are presented as means ± SEM. *n* = 10. ^****^p < 0.0001, control vs. db^−^/db^−^

To explore the potential mechanism of Piezo1 activation, we compared the total protein expression level of BK_Ca_ channel in the mesenteric arteries of control and db^−^/db^−^ mice. Our results showed no significant difference in the protein expression level of BK_Ca_ channel between the two groups (Fig. 7A and B). To investigate the concept of a co-localization between Piezo1 and the BK_Ca_ channel, we conducted double immunofluorescence staining followed by confocal analysis on the mesenteric arteries. Figure 7C illustrates that the staining for the anti-BK_Ca_ channel overlaps with that of anti-Piezo1, indicating the presence of Piezo1 and BK_Ca_ channel in very close spatial positions in both groups. Interestingly, the extent of co-localization was more pronounced in the db^−^/db^−^ mice (Fig. 7C and D). The statistical analysis indicated a significantly greater degree of co-localization in db^−^/db^−^ mice when compared to control mice. (Fig. 7E). The Pearson’s correlation coefficient of the two signals is 0.272 ± 0.024 and 0.536 ± 0.006 in control and db^−^/db^−^ mice, respectively, which indicates degree of correlation is higher in db^−^/db^−^ mice compared to control mice.

## Discussion

Our present study demonstrated that Piezo1 activation by its specific agonist, Yoda1, induced vasodilation in mesenteric resistance arteries in control and db^−^/db^−^ mice. This response was mediated through both endothelium-dependent and -independent mechanisms. In control mice, Yoda1-induced vascular relaxation was greater than in db^−^/db^−^ mice and was mainly endothelium dependent. Moreover, IK_Ca_, SK_Ca_, and BK_Ca_ channels are involved in Yoda1-induced relaxation in control mice. However, in db^−^/db^−^ mice, Yoda1-induced relaxation was slightly reduced by endothelial denudation and only BK_Ca_ channel blocker significantly reduced Yoda1-induced relaxation. Additionally, co-localization of BK_Ca_ and Piezo1 was increased in db^−^/db^−^ mice. These results indicated that Yoda1-induced vascular relaxation in db^−^/db^−^ mice is mainly caused by the BK_Ca_ channel present in vascular smooth muscle cells.

Endothelial dysfunction is a hallmark of diabetes and plays a pivotal role in the development of vascular complications [4]. In a previous study, we reported impaired endothelium-dependent relaxation (EDR) in mesenteric arteries of db^−^/db^−^ mice [3]. Here, we confirmed reduced ACh-induced EDR in mesenteric arteries of type 2 diabetic (db^−^/db^−^) mice compared to C57BL/6 (control) mice (data not shown). Piezo1 is a non-selective cation channel activated by mechanical stimuli including pressure, shear stress, and membrane tension [7]. This channel is permeable to cations, such as Na^+^, K^+^, Ca^2+^, and Mg^2+^, with a particular preference for Ca^2+^ [5]. Chemical activators such as Yoda1 are often used to investigate Piezo1 activation, as they have shown to open Piezo1 channels and promote Ca^2+^ influx flow across synthetic cell membranes [1]. Piezo1 plays a significant role in vascular tone regulation [11]. Activation of Piezo1 in the vasculature leads to an increase in intracellular calcium levels ([Ca^2+^]_*i*_), which subsequently activates endothelial nitric oxide synthase (eNOS) through specific signaling pathways. This cascade results in the production of endothelial nitric oxide (NO), leading vasodilation [1, 34]. However, despite the significance of Piezo1 in vascular function, to the best of our knowledge, there are no studies on the involvement of the Piezo1 in the mesenteric resistance artery of type 2 diabetic mice.

Our study revealed that concentration-dependent relaxation response induced by Yoda1 was reduced in the mesenteric resistance arteries in db^−^/db^−^ mice compared to the control mice (Fig. 2), which was associated with a reduction in Piezo1 expression level (Fig. 1). The specificity of Piezo1 activator, Yoda1, was supported by the substantial reduction in the relaxation response upon pre-treatment with the selective inhibitor Dooku1 (a selective inhibitor of Yoda1) and the mechanosensitive channel-selective inhibitor GsMTx4 (Fig. 4). However, Dooku1 and GsMTx4 did not completely eliminate Yoda1-induced relaxation. Our data was similar with the previous study reported by Evans E.L. et al. [10]. They described that Dooku1 effectively reduced Yoda1 activity by 60% in murine aortic rings. They also showed that Dooku1 did not completely block the intracellular Ca^2+^ increase induced by Yoda1 in human umbilical endothelial cells (HUVECs) while Dooku1 strongly inhibited Yoda1 in aorta. They suggested that Dooku1 is not perfect since it does not directly block the channels, but it is a new tool compound valuable for Piezo1 characterization studies. This is in accordance with our findings that Dooku1 antagonizes the action of Yoda1.

We further explored the mechanisms underlying Piezo1 activation-induced relaxation. The vascular endothelium, located between circulating blood and vascular smooth muscle, critically regulates vascular tone. Interestingly, removal of endothelium reduced relaxation responses induced by Yoda1 in both control and db^−^/db^−^ mice, indicating that endothelium contributes to Yoda1-induced relaxation (Fig. 2). However, the degree of reduction was significantly greater in control mice than in db^−^/db^−^ mice, which suggest that endothelium-dependent mechanism is more significantly involved in control mice than in db^−^/db^−^ mice. The endothelium is known to release a variety of vasodilator factors, such as nitric oxide (NO), prostacyclin (PGI_2_), and endothelium-derived hyperpolarizing factors (EDHF) in response to various stimuli [30]. Among them, NO is a potent endothelium-derived relaxing factor; it is synthesized from L-arginine and oxygen through the enzymatic action of endothelial nitric oxide synthase (eNOS). Subsequently, NO permeates into the vascular smooth muscle cells, initiating the activation of soluble guanylate cyclase (sGC) to generate guanosine 3′,5′-cyclic monophosphate (cGMP). This process results in the relaxation of the vascular smooth muscle [21, 23]. Our experiments demonstrated that pre-treatment with the NO synthase inhibitor (N^ω^-nitro-L-arginine, L-NNA) and the sGC inhibitor, 1H-[1,2,4]oxadiazolo [4,3,-a] quinoxalin-1-one (ODQ), attenuated Yoda1-induced relaxation in both groups, indicating the involvement of NO/cGMP signaling in this response (Fig. 4). However, we observed that the degrees of inhibitory effects of L-NNA and ODQ on Yoda1-induced relaxation are different in control mice. Since we used high concentration of ODQ (10^−5^ M) that sufficiently block sGC, we do not think this is due to the concentration. We assume that L-NNA may block vasorelaxation more significantly than ODQ because it inhibits the production of NO itself, impacting a broad range of NO-mediated processes and pathways (e.g., direct interaction with ion channels, protein modification through S-nitrosylation) [13, 22, 29]. In contrast, ODQ selectively inhibits sGC, specifically blocking the NO/cGMP pathway but leaving other NO-mediated effects intact. This broad-spectrum inhibition by L-NNA affects both direct and indirect actions of NO, leading to a more significant overall reduction in vasodilation. And in db^−^/db^−^ mice, because the contribution of endothelium and NO/cGMP in Yoda1-induced relaxation is much smaller than in control mice, this effect is not observed.

Besides NO, another essential vasodilator produced by the endothelium is PGI_2_, a prostanoid synthesized from arachidonic acid through enzymatic activity in the cyclooxygenase (COX) pathway. PGI_2_ promotes relaxation of vascular smooth muscle by activating adenylate cyclase, which leads to the production of adenosine 3′,5′-cyclic monophosphate (cAMP). This cAMP then reduces intracellular Ca^2+^ levels, resulting in the relaxation of vascular smooth muscle [32, 33]. However, pre-treatment with the cyclooxygenase (COX) inhibitor, indomethacin, did not have a significant effect, suggesting that PGI_2_ production may not be a major contributor (Fig. 4).

Additionally, vascular tone is modulated by regulation of K^+^ channels, including Ca^2+^-activated K^+^ channels (K_Ca_). Within the vascular wall, there are three subtypes of calcium-activated potassium channels (K_Ca_): large, intermediate, and small conductance (BK_Ca_, IK_Ca_, and SK_Ca_). Intriguingly, our observations revealed that IK_Ca_ channel blocker (TRAM-34) and SK_Ca_ channel blocker (apamin) partially inhibited Yoda1-induced relaxation in control mice. This suggests the involvement of IK_Ca_ and SK_Ca_ channel in Yoda1-induced relaxation. However, in db^−^/db^−^ mice, no significant effect was observed after pre-treatment with TRAM-34 and apamin (Fig. 5). In order to elucidate the involvement of BK_Ca_ channel, we treated the BK_Ca_ channel blocker, ChTX, to endothelium-intact and -denuded arteries. Treatment of ChTX reduced Yoda1-induced relaxation in endothelium-intact arteries in both control and db^−^/db^−^ mice. Interestingly, in endothelium-denuded arteries, Yoda1-induced relaxation was markedly inhibited by ChTX only in db^−^/db^−^ mice (Fig. 6). Overall, we observe a mild effect of IK_Ca_ and SK_Ca_ inhibitors on Yoda1-induced relaxation in control mice, which is lost in db^−^/db^−^ mice, and a greater effect of the BK_Ca_ channel inhibitor on db^−^/db^−^ arteries as compared to control mice. Since IK_Ca_ and SK_Ca_ channels are predominantly expressed in the endothelial compartment and BK_Ca_ channels are mainly expressed in smooth muscle [12], it could be explained well with the expectation that smooth muscle cells play a significant role in Yoda1-induced relaxation in db^−^/db^−^ mice which exhibit endothelial dysfunction. Furthermore, our findings are in accordance with the previous study shown that inactivation of endothelial IK_Ca_ and SK_Ca_ channels contributes to coronary arteriolar dysfunction in diabetic patients [18]. They reported that diabetes significantly decreased endothelial hyperpolarization and SK _Ca_/IK _Ca_ currents induced by the SK _Ca_/IK_Ca_ activator, NS309, as compared with that of nondiabetics.

Despite the low expression of Piezo1 in db^−^/db^−^ mice as shown in Fig. 1 and no difference in the BK_Ca_ channel expression (Fig. 7A and B), decrease in Yoda1-induced relaxation by ChTX treatment was greater in db^−^/db^−^ mice. Therefore, we assume that the association between the BK_Ca_ channel and Piezo1 would be more pronounced in db^−^/db^−^ mice. The immunofluorescence staining and confocal analysis data further supported this intriguing concept that Piezo1 and BK_Ca_ channel exist closer in db^−^/db^−^ mice (Fig. 7C–E). It is thought that increase in co-localization of Piezo1 and BK_Ca_ may serve as a compensatory mechanism in response to the reduced Piezo1 expression observed in db^−^/db^−^ mice. A previous study has shown that BK_Ca_ channel mechano-sensitivity partially depends on stretch-activated Ca^2+^ influx via Piezo1 in human atrial fibroblasts [15]. They suggested that Piezo1 and BK_Ca_ channels are functionally linked. Furthermore, our data are supported with another previous finding that in human glioblastoma cells, Yoda1 activates both IK_Ca_ and BK_Ca_ currents, which shows that these channels are under the control of Piezo1 [19, 20].

The present study is the first report on the distinct aspects of vascular response induced by Piezo1 activation in the mesenteric resistance arteries of db^−^/db^−^ mice. Physiologically, Piezo1 is predominantly found in endothelial cells and plays a role in promoting vascular relaxation. However, in pathological situations such as diabetes, endothelial cell damage may cause up-regulation of Piezo1 in smooth muscle cells, aiding in blood vessel relaxation. This compensatory mechanism likely contributes to maintain some degree of blood vessel relaxation in diabetes. Our findings could provide novel insights for identifying the potential mechanisms contributing to vascular dysfunction in diabetes.

## Data Availability

All data supporting the findings of this study are available within the paper

## Abbreviations

ACh: Acetylcholine
SNP: Sodium nitroprusside
NO: Nitric oxide
sGC: Soluble guanylyl cyclase
COX: Cyclooxygenase
L-NNA: N^ω^-nitro-L-arginine
ODQ: 1H-[1,2,4]oxadiazolo [4,3,-a] quinoxalin-1-one
INDO: Indomethacin
ChTX: Charybdotoxin
BK_Ca_: Large-conductance Ca^2+^-activated K^+^ channels
TRAM-34: Rriarylmethane-34
IK_Ca_: Intermediate-conductance Ca^2+^-activated K^+^ channels
SK_Ca_: Small-conductance Ca^2+^-activated K^+^ channels
DMSO: Dimethyl sulfoxide
cGMP: Guanosine 3′,5′-cyclic monophosphate
cAMP: Adenosine 3′,5′-cyclic monophosphate
W/O: Wash out
SEM: Standard error of the mean
ANOVA: Analysis of variance
PCC: Pearson’s correlation coefficient
